# Intraluminal Gossypiboma

**Published:** 2014-05-21

**Authors:** Yousuf Aziz Khan, Muhammad Asif, Wasmi Al-Fadhli

**Affiliations:** Ibn Sina Specialized Surgical Hospital, Al-Sabah Health Region, Kuwait.

**Keywords:** Gossypiboma, Retained surgical sponge, Children, Thalassemia, Splenectomy

## Abstract

Gossypiboma (GP) or retained surgical sponge is one of the rare surgical complications which can happen despite precautions. Because of the medico-legal issues, it is under-reported. An 8-year-old thalassemic girl, with a history of splenectomy and cholecystectomy, presented to us with acute intestinal obstruction and required surgical exploration. Intraluminal gossypiboma obstructing the ileum was found. Though a rare cause, gossypiboma should also be included in the differential diagnoses of postoperative intestinal obstruction.

## INTRODUCTION

A foreign body may be left inadvertently at any site and during any surgical procedure. Gossypiboma (GP), the term derived from ‘gossypium’ (Latin) meaning cotton and ‘boma’ (Swahili) meaning place of concealment, usually refers to a retained surgical sponge.[1] The exact incidence of this rare surgical complication is not known because of being under-reported. However, some have mentioned an incidence of 1 among 100-15,000 abdominal surgical procedures.[1,2] Mostly GPs have been described after abdominal surgeries, however, GP at other sites such as neck, thorax and thigh have also been reported.[1] Cholecystectomy, appendectomy, and hysterectomy are common surgical procedures with a high occurrence of GPs probably due to high number of procedures done worldwide.[3] It may present in the early post-operative period or years later after surgery, or remain asymptomatic and diagnosed incidentally.[3-5] Trans-mural migration of a GP is a rare consequence of intra-peritoneal GPs.[6] Very rarely, GP are reported in the pediatric population. Herein, we report a case of intraluminal GP in a thalassemic girl.

## CASE REPORT

An 8-year-old girl, known case of thalassemia major, was referred to us from a private center with the diagnosis of acute intestinal obstruction. She had open splenectomy and cholecystectomy in a hospital at another country two years back. She developed multiple episodes of vomiting, initially non-bilious and later bilious, and abdominal pain for two days prior to presentation. On examination, she was an ill looking, pale girl with a heart rate of 120/min, temperature of 37.4°C and BP of 106/69 mmHg. There was a midline supra-umbilical scar. Abdominal fullness and moderate diffuse tenderness was found at examination. Laboratory workup revealed Hb of 7.7 gm/dl, TLC of 21.7×109/L, and platelets count of 994×109/L. Serum electrolytes were deranged with a serum Na+: 127 mmol/L and serum Cl- : 92 mmol/L. Her coagulation profile was also deranged with a raised Prothrombin time of 19.3 with an INR of 1.485. Air fluid levels were noticed on erect abdominal X-ray. Ultrasound abdomen and pelvis reported dilated fluid filled bowel loops, mild free fluid in peritoneal cavity, and a blind ending aperistaltic, non-compressible structure in the right lumbar region with positive probe tenderness; a suspicion of appendicitis was raised. She had contrast enhanced CT scan of abdomen and pelvis already with her which showed a circumferentially thick dilated ileal loop with focal enhancement containing an ill-defined heterogeneous lesion with spongiform appearance (Fig.1, 2).

**Figure:**
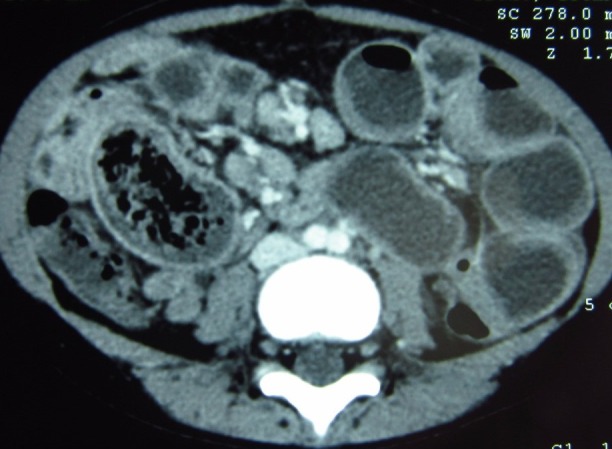
Figure 1: CT scan abdomen showing dilated fluid filled bowel loops with a heterogeneous mass in the small bowel lumen with spongiform appearance.

**Figure:**
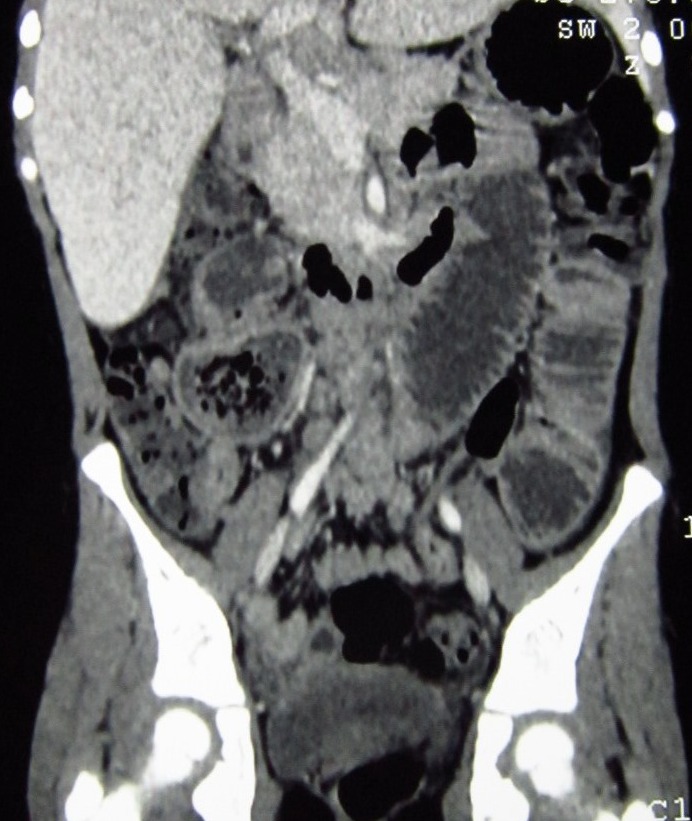
Figure 2: Coronal view

After optimization, an initial attempt was done laparoscopically but the procedure had to be converted to open via the previous midline supra-umbilical scar due to bowel distension and dense adhesions. There were dense adhesions between transverse colon, omentum and liver. The mid-ileal loop was densely adherent at the gall bladder bed; when mobilized it was found to have an old sealed perforation. Some iatrogenic perforations also occurred while mobilizing adherent ileum and, resection and primary anastomosis was performed. The resected ileal segment contained an old retained surgical gauze piece inside the lumen (Fig. 3). Post-operative course was difficult as she had persistent high grade fever and evidence of urinary tract infection but settled with antibiotics coverage. She was orally allowed on 6th and discharged home on 11th post-operative days, respectively. After 10 days she was followed up in the out-patient clinic and was doing fine without any complication.

**Figure:**
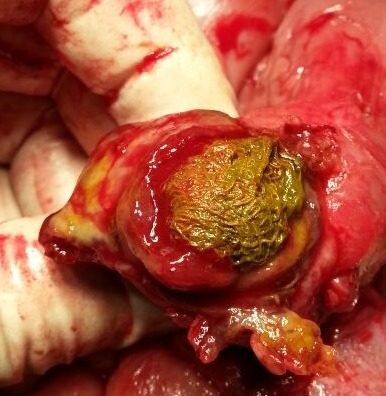
Figure 3: Showing a completely intraluminal gossypiboma in the ileal loop.

## DISCUSSION

Surgical sponges are the most frequent gossypibomas (GP) because of their common use, size and consistency.[7] Two types of body responses are induced by a GP: an aseptic fibrinous response causing adhesions, encapsulation and a resulting foreign body (FB) granuloma; and an exudative response that leads to development of abscess and, internal or external fistula formation.[3,4,8] The clinical presentation is variable depending upon the type of body response to a GP. Complete transmural migration of a GP is rarely reported.[6,7,9] An unintentionally left surgical gauze at the gall bladder bed during previous cholecystectomy caused an inflammatory response and resultant inflammation and adhesion of the ileal loop, that led to necrosis and subsequent transmural migration of gauze into ileum causing obstruction, is the possible explanation of the presentation of GP in our case.

Once diagnosed a GP should be removed as early as possible to evade any added surgical morbidities and both open and minimally invasive procedures (endoscopy, laparoscopy) have been described.[3,5,6] A meticulous count of the surgical material pre, intra and post-operatively, systematic check of the surgical site before concluding a procedure, only use of sponges impregnated with a radio-opaque marker and radiologic screening in case of doubt in high risk scenarios, are some of the gold standard practices.[2,5,7,9,10] New technologies as radiofrequency identification (RFID) system have also been introduced to minimize this complication.[6,7]

In summary, a high index of suspicion is required to diagnose a GP presenting long time after first surgery. Standard safety practices should always be followed to prevent this rare surgical complication.

## Footnotes

**Source of Support:** Nil

**Conflict of Interest:** None declared

